# A cost-sensitive multiclass machine learning framework for postoperative neurosurgical triage (Neuro-TACTIC)

**DOI:** 10.1038/s41598-026-45092-1

**Published:** 2026-03-24

**Authors:** Paul Vincent Naser, Maximilian Fischer, Roberto Diaz Peregrino, Martin Jakobs, Sandro Krieg, Peter Neher, Jan-Oliver Neumann

**Affiliations:** 1 Department of Neurosurgery, Heidelberg University Hospital, Im Neuenheimer Feld 400, 69120 Heidelberg, Germany; 2 Medical Faculty, Heidelberg University, Grabengasse 1, 69117 Heidelberg, Germany; 3 Division of Medical Image Computing, Germany, German Cancer Research Center (DKFZ) Heidelberg, Im Neuenheimer Feld 280, 69120 Heidelberg, Germany; 4 Division of Stereotactic Neurosurgery, Department of Neurosurgery, Heidelberg University Hospital, Im Neuenheimer Feld 400, 69120 Heidelberg, Germany; 5 German Cancer Consortium (DKTK), Partner Site Heidelberg, Heidelberg, Germany; 6 National Center for Tumor Diseases (NCT), NCT Heidelberg, a Partnership Between DKFZ and the University Medical Center Heidelberg, 69120 Heidelberg, Germany; 7 Pattern Analysis and Learning Group, Department of Radiation Oncology, Heidelberg University Hospital, 69120 Heidelberg, Germany; 8 German Cancer Research Center (DKFZ), AI Health Innovation Cluster, Heidelberg, Germany

**Keywords:** Neurosurgical triage, Intensive care unit, Neurosurgical intensive care, Cost-sensitive classification, Machine learning, Artificial intelligence, Health care, Medical research, Neurology, Oncology

## Abstract

Postoperative placement of patients into a regular ward, an intermediate-care unit (IMC), or an intensive care unit (ICU) is critical for balancing patient safety against resource constraints. Most existing models collapse this decision into a binary ICU versus non-ICU choice and lack a mechanism to tune risk thresholds to local staffing ratios or definitions of ICU‐level events. We developed Neuro-TACTIC, a cost-sensitive machine learning framework that stratifies postoperative neurosurgical patients into three monitoring levels: regular ward, intermediate care unit, and intensive care unit. An XGBoost-based classifier was trained on 27 demographic, intraoperative, and imaging-derived features from a retrospective cohort of 1072 patients undergoing elective craniotomy. A tunable parameter ζ integrates resource-related and harm-related costs to adjust the balance between over- and under-triage. Generalization was assessed in an independent cohort. Across repeated cross-validation and bootstrap analyses, the framework demonstrated stable behavior across cost settings. At the operating point ζ = 0.975, performance was AUCμ = 0.67 ± 0.03 and weighted F1 = 0.49 ± 0.03 in the development cohort, and AUCμ = 0.60 ± 0.04 and weighted F1 = 0.44 ± 0.06 in the independent evaluation cohort (n = 81). Feature importance analyses identified operative duration, tumor volume, surgical position, body mass index, and patient age as the most influential predictors. This study demonstrates the feasibility of cost-sensitive, three-tier postoperative triage modeling in neurosurgical patients. Neuro-TACTIC is a methodological proof-of-concept; prospective validation and multicenter evaluation are required before clinical deployment.

**Supplementary Information:**

The online version contains supplementary material available at 10.1038/s41598-026-45092-1.

## Introduction

Postoperative patient allocation to appropriate monitoring levels is a fundamental challenge in neurosurgical care. Decisions regarding placement on a regular ward, an intermediate care unit (IMC), or an intensive care unit (ICU) must balance early detection of deterioration with finite staffing and bed resources. Overly conservative triage increases resource utilization, whereas insufficient monitoring risks delayed recognition of adverse events^1–3^.

Neurosurgical patients represent a particularly demanding population for postoperative triage. Intracranial procedures carry risks such as hemorrhage, cerebral edema, and acute neurological decline, which may evolve rapidly and require frequent neurological assessment. As a result, many institutions default to routine ICU admission after elective craniotomy, despite longstanding evidence that only a subset of patients require ICU-level interventions^4–7^.

Prior efforts to formalize postoperative neurosurgical triage have primarily relied on binary classification schemes and regression-based risk scores (ICU vs. non-ICU). For example, in an attempt to identify preoperative factors associated with postoperative ICU-requiring events, Hanak et al. examined 400 elective craniotomy cases and, using multivariate analysis, identified factors predictive of postoperative ICU admission. Several other tools and scores have been developed to assess the risk of postoperative ICU-level care in neurosurgical patients, analyzing parameters such as ASA score, tumor volume, and surgical duration, and reporting ROC-AUC values between 0.7 and 0.77^1,2^, which could be validated in studies on novel patient cohorts^9,10,33^.

However, existing approaches share important limitations. By collapsing postoperative care into a binary ICU versus non-ICU decision, they fail to account for the growing role of intermediate care units^8^, which serve patients who require closer monitoring than a ward but do not meet criteria for full ICU support. Moreover, these models do not explicitly account for the asymmetric consequences of misclassification, and they typically optimize for statistical performance metrics rather than operational or resource-aware consideration^1,2,9,10^.

**Fig. 1:**
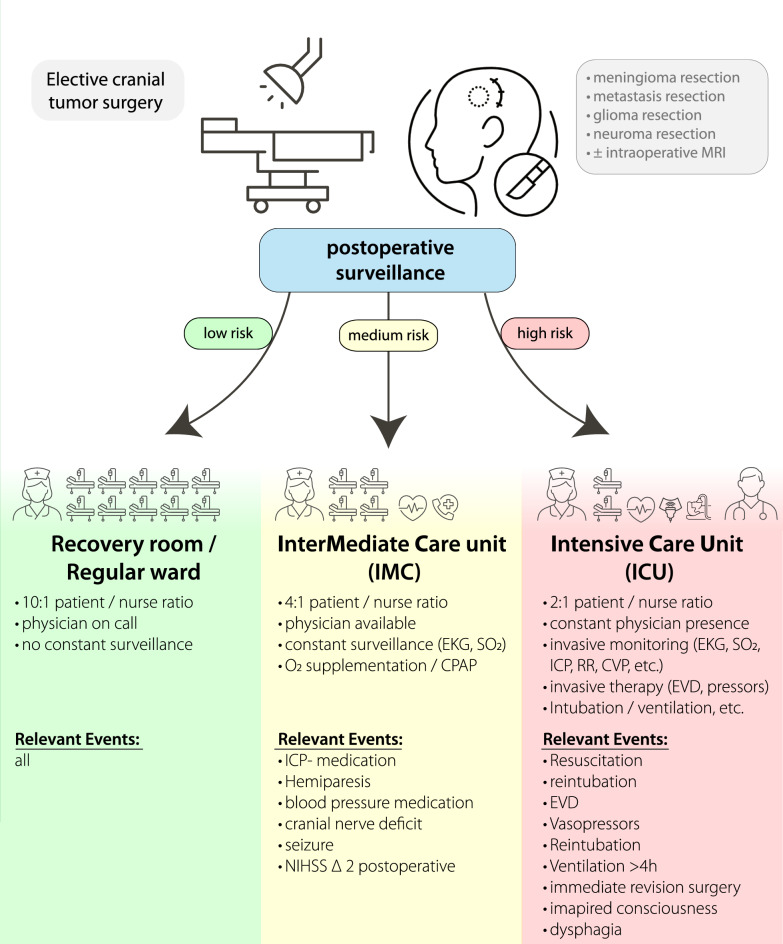
Following elective cranial tumor surgery, patients undergo early postoperative surveillance to assess their risk of neurologic or hemodynamic deterioration. Based on their risk profile framework, patients are stratified into three acuity tiers, low risk (green, left), medium risk (yellow, middle), or high risk (red, right)—and allocated accordingly to: Recovery room / Regular ward (left, 10:1 patient-to-nurse ratio) reserved for low-risk patients requiring only routine vital‐sign monitoring and on-call physician coverage. Intermediate Care Unit (middle, IMC; 4:1 ratio): assigned to medium‐risk patients needing continuous surveillance (EKG, SpO₂), on-site physician availability, noninvasive support (oxygen supplementation, CPAP), and prompt management of events such as intracranial-pressure–lowering medications, new cranial‐nerve deficits, seizures, or a ≥ 2‐point rise in NIHSS. Intensive Care Unit (right, ICU; 2:1 ratio): reserved for high‐risk patients who require constant physician presence, invasive monitoring (ICP, CVP, arterial lines), advanced therapies (EVD, vasopressors), mechanical ventilation, and rapid intervention for critical events (resuscitation, reintubation, immediate surgical revision, impaired consciousness, dysphagia).

Recent advances in machine learning enable more flexible modeling of complex perioperative data to improve neurosurgical care^9–14^, and boosted decision trees have been shown to outperform traditional regression-based statistical methods^10^. Nevertheless, to date, no published framework integrates cost sensitivity or provides a transparent mechanism to adapt triage thresholds to local resource constraints and institutional risk tolerance. To address this gap, we propose Neuro-TACTIC (**Neuro**surgical **T**riage & **A**cuity algorithm via **C**ost-**T**uned **I**CU/IMC **C**lassification). This cost-sensitive machine learning framework models postoperative triage as a three-class problem and introduces a tunable parameter (ζ) to balance harm and resource considerations (Fig. 1). In this proof-of-concept study, we aim to develop this three-tiered classification paradigm and internally and externally evaluate the algorithm as a precursor to further clinical studies.

## Methods

### Patient population

For the main dataset, 1072 consecutive adult patients admitted to our ICU following elective craniotomy at our institution between 01/2019 and 07/2020 were included in the study. Parts of this dataset have been previously analyzed in the context of a binary ICU/non-ICU decision paradigm^9,10^. For the evaluation dataset, 81 adult patients undergoing elective brain tumor surgery in 11–12/2024 were consecutively enrolled (Table 1). Emergency cases were excluded. Overall, the majority of patients did not require either ICU or IMC surveillance (62.9% in the main dataset, 67.1% in the evaluation dataset; Tables 2, 3).

**Table 1:** Overview of demographic features of patients included in the analysis. The chi-square test was used to assess categorical variables with more than two categories, in which case the resulting p-value is denoted in the first row relating to that category. For binary variables, Fisher’s exact test was used, and unpaired t-test with Welsh’s correction for continuous variables.

Feature	Main dataset n (%)	Evaluation dataset n (%) / mean ± SD	*p*-value
Total patients	1072	81	
Age (years)	56.85 ± 14.71	56.68 ± 15.72	0.9260
ASA			0.7647
I	59 (5.5%)	3 (3.7%)	
II	629 (58.7%);	45 (55.6%)	
III	375 (35.0%)	32 (39.5%)	
IV	9 (0.8%)	1 (1.2%)	
BMI	26.53 ± 4.96	27.18 ± 6.136	0.3633
Sex			0.3119
Female	606 (56.5%)	51 (63.0%)	
Male	466 (43.5%)	30 (37.0%)	
Weight	77.91 ± 17.00	78.30 ± 20.60	0.8694
Glasgow Coma Scale			< 0.0001
15	1051 (98.0%)	69 (85.2%)	
14	18 (1.7%)	8 (9.9%)	
11	3 (0.3%)	0 (0.0%)	
Preoperative Neurological deficit	643 (60.0%)	56 (69.1%)	0.1315
History of seizures	263 (24.5%)	13 (16.0%)	0.1117
Hypertension	450 (42.0%)	38 (46.9%)	0.4530
Coagulation disorder	21 (2.0%)	0 (0.0%)	0.4007
Diabetes	121 (11.3%)	8 (9.9%)	0.8371
Preexisting other cardiovascular disease e.g. myocardial infarction, coronary heart disease, aFib etc	143 (13.3%)	24 (29.6%)	< 0.0001
Preexisting pulmonary disease, e.g. COPD, asthma,	484 (45.1%)	31 (38.3%)	0.2299
Other preexisting diseases e.g. renal insufficiency	152 (14.2%)	19 (23.5%)	0.0235

**Table 2:** Overview of tumor-relevant features extracted from the preoperative radiology report. The chi-square test was used to assess categorical variables with more than two categories, in which case the resulting *p*-value is denoted in the first row relating to that category. For binary variables, Fisher’s exact test was used, and unpaired t-test with Welsh’s correction for continuous variables.

Feature	Main dataset n (%)	Evaluation dataset n (%)	*p*-value
Tumor volume	21.47 ± 31.23	19.82 ± 27.21	0.6068
Hydrocephalus on MRI	61 (5.7%)	3 (3.7%)	0.6162
Tumor location			0.002
Intratentorial	251 (23.4%)	33 (40.7%)	
Supratentorial	789 (73.6%)	48 (59.3%)	
Midline shift (> 3 mm) on MRI	186 (17.4%)	21 (25.9%)	0.0736
Suspected diagnosis			0.0001
Meningeoma	364 (34.0%)	31 (38.3%)	
Metastasis	188 (17.5%)	18 (22.2%)	
LGG	74 (6.9%)	2 (2.5%);	
HGG	39 (3.6%)	3 (3.7%)	
GBM	164 (15.3%)	11 (13.6%)	
Neuroma (including vestibular)	75 (7%)	0 (0.0%)	
Haemangioblastoma	1 (0.09%)	0 (0.0%)	
Other	167 (15.6%)	14 (17.3%)

**Table 3:** Overview of ICU and IMC events in the main and evaluation datasets. Fisher’s exact test was used to test for significant differences between groups.

	Event	Main dataset n (%)	Evaluation dataset n (%)	*p*-value
ICU-events	Cardiopulmonary resuscitation	4 (0.4%)	0 (0.0%)	1.0000
	Drainage of cerebrospinal fluid	22 (2.1%)	1 (1.2%)	1.0000
	Immediate operative revision	13 (1.2%)	1 (1.2%)	1.0000
	Catecholamine administration	24 (2.2%)	0 (0.0%)	0.4057
	Reintubation	12 (1.1%)	2 (2.5%)	0.2576
	Dysphagia	18 (1.7%)	0 (0.0%)	0.6313
	Mechanical ventilation > 4h	33 (3.1%)	0 (0.0%)	0.1629
	Impaired consciousness	35 (3.3%)	4 (4.9%)	0.3467
IMC-events	i.v.-antihypertensive drug application	252 (23.5%)	6 (7.4%)	0.0004
	Cranial nerve deficit	74 (6.9%)	18 (22.2%)	0.0000
	Severe hemiparesis	48 (4.5%)	0 (0.0%)	0.0437
	Medication to lower ICP	49 (4.6%)	0 (0.0%)	0.0437
	mNIHSS-score worsened by 2 points	75 (7.0%)	0 (0.0%)	0.0079
	Seizure	43 (4.0%)	0 (0.0%)	0.0676
	≥ 1 IMC-event, no ICU event	265 (24.7%)	21 (25.9%)	0.7906
	≥ 1 ICU event	133 (12.4%)	6 (7.4%)	0.2172
	Neither ICU nor IMC event	674 (62.9%)	54 (66.7%)	0.6322

Our in-house protocol does not mandate routine ICU surveillance after surgery. The decision for postoperative surveillance in the ICU was made prior to surgery by joint judgment of the neurosurgeon and the anesthesiologist.

### Data collection

Data relevant to the study were retrospectively extracted from clinical records, and preoperative imaging was reviewed. The anesthesia report and subsequent ICU and neurosurgical floor documentation were scrutinized for any intra- or postoperative adverse events. A routine postoperative CT scan is not performed at our institution. There was no missing data in our study.

### Prognostic features

Based on the literature^1,2,9,10^, we selected candidate features from five categories that were potentially prognostic for predicting adverse events. Table 1 summarizes these medical features, while Table 2 presents the imaging-derived features.

### Postoperative events and levels of surveillance

Based on the literature^1,2,9,10^, we defined events and interventions that require treatment in an ICU setting (Table 3). We also recorded other events that require specific actions or an elevated level of staff attention and defined these as IMC events (Table 3). The ICU was defined as the highest level of care, capable of managing both ICU and IMC events, whereas IMC could only successfully treat IMC events. The third option, the ward, was defined as incapable of treating either IMC or ICU events.

### Training and validation of machine learning algorithms

For the classification task, a supervised gradient boosting technique was used. Gradient boosting uses an ensemble of decision trees that are iteratively optimized to minimize the loss function. The XGBoost framework (V1.7.6) was selected^15^. Boosted trees were trained, optimized, and their final performance validated using fivefold cross-validation repeated 5 times for a total of 25 training and validation runs (training set: 800 cases, validation set: 200 cases). Hyperparameters were optimized using fivefold cross-validation on each training set with the Optuna framework (V3.3.0) and a tree-structured Parzen estimator, with 5000 iterations per run. Additional ML tasks were performed with the scikit-learn^15^ package (V1.2.2)^32^.

### Integration of a cost-matrix and a weighted loss function

To incorporate domain-specific misclassification costs into model training, we implemented a custom multiclass classification function in XGBoost. We defined a cost-sensitive loss based on a class-dependent cost matrix that specifies penalties for misclassifying each class. This matrix was parameterized by a tunable $\zeta$ value to adjust the relative severity of the different errors. During training, we implemented a custom gradient and Hessian computation, which modifies the optimization process to minimize the expected misclassification cost rather than the nominal classification error. This allowed the model to prioritize clinically relevant distinctions, particularly when misclassifying certain classes (e.g., ICU) has a higher practical impact. The use of custom objectives in XGBoost builds upon its native support for user-defined loss functions through second-order gradient optimization^15^. The mathematical details are explained in the supplementary methods.

### Classification performance with HC and RC

In our model, the trade-off between staffing resources and under-triage risk is mediated by the factor ζ, which determines the relative weight of cost considerations in this relationship. On one hand, the relative cost matrix (RC) operationalizes nurse-to-patient ratios (ward 10:1, IMC 4:1, ICU 2:1) as a transparent proxy for relative nursing resource intensity and is row-normalized. This choice is supported by the health services literature, in which nursing coverage/workload is explicitly used as a proxy for ICU resource utilization^16^. Moreover, nursing staffing constitutes a major component of ICU costs and has been linked to workload-based resource planning approaches^17^. RC is not intended to represent total economic cost, which also depends on physician staffing, monitoring infrastructure, medication use, institutional accounting, and opportunity costs; these components would require institution-specific microcosting approaches, for which nursing activity measures have been proposed as key inputs^18^.

On the other hand, the harm-cost matrix (HC) weights under-triage errors using inverse class frequencies in the development cohort to penalize misclassification of rare ICU/IMC outcomes more strongly. This frequency-based weighting is a pragmatic proxy for unequal consequences of misclassification and should not be interpreted as a direct measure of clinical severity. The final cost matrix was computed as a ζ-weighted combination of RC and HC (full equations in Supplementary Methods).

Practical guidance for institution-specific calibration of ζ (data requirements, validation workflow, and ζ-sensitivity analysis) is provided in the Supplementary Methods.

### Cross-validation and performance evaluation

We evaluated our classifier on both an internal and an external hold-out dataset using a repeated stratified cross-validation paradigm. After training, we computed probabilistic predictions on the held-out test fold and on the entire external cohort. We quantified discrimination using the area under the cost-weighted multiclass ROC surface (AUCµ)and the class-imbalance-aware F_1_ score^30,31^. Specifically, we recorded both a single weighted F_1_ (averaging per-class F_1_ by ground-truth support) and the individual F_1_ for each of the three outcome classes. Confusion‐matrix counts and runtimes were also logged. All fold‐level metrics were exported for downstream summary and statistical comparison across $\zeta$ settings. For external evaluation, each of the 25 trained cross-validation models was applied to the independent evaluation cohort, yielding a distribution of external-cohort performance metrics across models; we report the mean ± SD and 95% confidence interval for the mean across these 25 model evaluations.

### Feature importance

In our analysis, we used two complementary approaches to assess feature importance from the trained XGBoost models: gain-based importance and SHAP values.

Gain importance reflects the total gain in the objective function brought by each feature when it is used in a decision tree split across all boosting rounds. Specifically, we extracted gain values via model.get_score(importance_type = ‘gain’), which provides a global measure of feature utility based on the training data and the structure of the fitted trees.

To complement this with a more individualized and model-agnostic explanation, we computed SHAP values^19^. SHAP is a unified framework grounded in cooperative game theory that attributes a contribution value to each feature for a given prediction. For multiclass outputs, SHAP produces class-specific explanations that we aggregated across classes and samples by averaging the absolute SHAP values, yielding a robust per-feature importance estimate tailored to the input data. This dual approach provides both a training-time (gain) and a prediction-time (SHAP) perspective on the relevance of each input feature.

### Statistics

Data were collected and processed in Microsoft Excel (Microsoft Corp. CA, USA) and submitted to descriptive and statistical analysis using GraphPad Prism (version 10.1.0; GraphPad Software LLC, Boston/MA, USA) and the scipy package (version 1.11.2) ^20,21^.

The comparison between ratios of occurrence relied on Chi-square testing for categorical variables with more than two values, and Fisher’s exact test for 2 × 2 comparisons. Comparing two individual groups of continuous variables relied on two-sided Student t-tests. In all cases, a p-value < 0.05 was considered statistically significant. Additional details about the statistical analysis are provided in the figure legends. Absolute values are provided as mean ± standard deviation (s.d.), unless otherwise noted in the figure legends.

For figure design, bar graphs, box plots, and other data illustrations were exported as vector graphics from Python and GraphPad Prism, and were arranged in Adobe Illustrator and Photoshop (Adobe Inc., San Jose, CA, USA).

### Ethics approval

The study was performed in accordance with the Declaration of Helsinki. The design of this study, as well as the retrospective collection and analysis of patient data, were approved by the institutional review board. The requirement of informed consent for collection and processing of pseudonymized patient data was waived.

## Results

### Demographics and comorbidities

The main cohort comprised 1072 patients, with an independent evaluation sample of 81 patients. Demographics and comorbidities were similar between cohorts (Table 1), except for higher rates of cardiovascular and other diseases in the evaluation set (*p* < 0.001; *p* = 0.0235). These distribution shifts indicate a measurable change in case mix between cohorts and motivate a cautious interpretation of external performance estimates, particularly for the rarer ICU class.

### Tumor-related features

Radiological and tumor-related features likewise showed substantial concordance (Table 2). Mean tumor volume did not differ (21.5 ± 31.2 vs. 19.8 ± 27.2 mL, *p* = 0.607), nor did rates of hydrocephalus (5.7% vs. 3.7%, *p* = 0.616) or midline shift (17.4% vs. 25.9%, *p* = 0.074). However, tumor location was more often supratentorial in the main cohort (73.6% vs. 59.3%, *p* = 0.002), and the distribution of suspected diagnoses differed (*p* = 0.0001), with fewer vestibular schwannomas and low-grade gliomas in the evaluation data.

### ICU and IMC events

Postoperative ICU and IMC events were generally rare and occurred at a similar frequency (Table 3). Regarding the ICU events, rates of cardiopulmonary resuscitation, cerebrospinal fluid drainage, immediate operative revision, catecholamine use, reintubation, dysphagia, prolonged mechanical ventilation, and impaired consciousness showed no significant differences between groups except for “medication to lower intracranial pressure,” which occurred in none of the evaluation patients (*p* = 0.0437). Among IMC events, IV antihypertensive administration (23.5% vs. 7.4%, *p* = 0.0004), new cranial nerve deficits (6.9% vs. 22.2%, *p* < 0.0001), severe hemiparesis (4.5% vs. 0%, *p* = 0.0437), and worsening mNIHSS by ≥ 2 points (7.0% vs. 0%, *p* = 0.0079) were more frequent in the main cohort. At the same time, seizures did not differ significantly (*p* = 0.0676).

The proportions of patients classified as requiring IMC (i.e., experiencing at least one IMC event without ICU transfer) versus those classified as requiring ICU care (i.e., experiencing at least one ICU event) were similar (*p* = 0.79 and *p* = 0.22, respectively).

### Classification performance

**Fig. 2:**
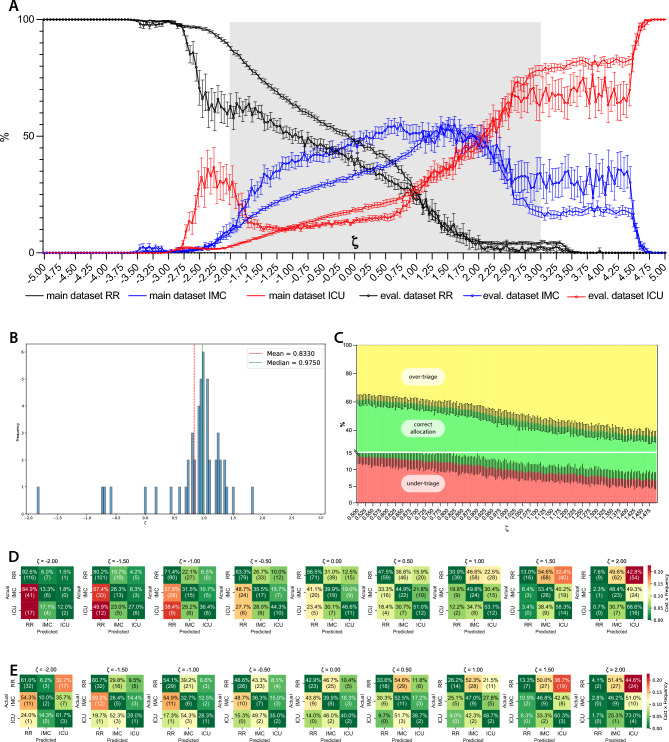
Cost‐sensitive triage performance and optimal ζ selection. (**A**) Fractions of patients assigned to regular ward (black), intermediate‐care unit (IMC; blue), or intensive care unit (ICU; red) as the cost‐sensitivity parameter ζ varies from -5 to + 5 in increments of 0.1. Solid lines denote the development cohort (n = 1072), and lighter markers with error bars denote the independent evaluation cohort (n = 81). When ζ is highly negative, prohibitive over‐triage costs drive almost all assignments to the ward; as ζ increases, IMC assignments peak in the shaded “tuning” region (ζ≈0.5–1.5), before ICU allocations dominate at high ζ. Error bars represent ± s.d. across 25 cross‐validation folds. (**B**) Histogram of ζ inflection points—defined as the ζ value at which the derivative of the total over‐triage rate reaches its maximum—across 50 bootstrapped cross‐validation runs. The median (green dashed line, 0.975) and mean (red dashed line, 0.833) are indicated. Clinically, this inflection point serves as an operating-point heuristic within the tuning range: it marks the region where further increases in ζ begin to yield diminishing reductions in under-triage while disproportionately increasing over-triage (i.e., escalation to higher-acuity care), reflecting a practical “diminishing returns” trade-off between under-triage risk and resource use. (**C**) Stacked plot of under‐triage (red), correct allocation (green), and over‐triage (yellow) rates as functions of ζ, illustrating how the balance of error types shifts smoothly across the tuning range. (**D, E**) Nine‐cell confusion matrices for representative ζ settings (–2.0, –1.5, –1.0, –0.5, 0.0, + 1.0, + 1.5, + 2.0) in (**D**) the development cohort and (**E**) the evaluation cohort. In each matrix, rows correspond to the true acuity class (ward, IMC, ICU), and columns correspond to the predicted class; cell shading reflects the proportion of patients in each category. At ζ = –2.0, under-triage (lower-left cells) is maximal, whereas at ζ =  + 1.0, diagonal dominance indicates balanced, majority-correct triage in both datasets. Detailed confusion matrices for the main and evaluation cohorts are further supplied in supplementary Figures S1 and S2.

To assess the model’s optimal performance, we performed a course sweep over ζ from − 5 to + 5 and recorded the resulting fractions of patients allocated to the three classes (Fig. 2A). For both the main and the independent evaluation cohort, the curves trace a smooth transition: at ζ≈ − 5 the model sends almost all patients to the ward, since any upward reclassification would incur prohibitive resource‐use penalties; as ζ increases toward zero, the penalty for under‐triage grows relative to resource cost, and the IMC curve rises first, peaking around ζ≈ + 0.5 where a roughly equal mix of ward and IMC assignments maximizes balanced coverage. Beyond ζ≈ + 1.0, the ICU curve ascends steeply: at these settings, the harm‐cost term dominates, so that even marginal cases are escalated. Crucially, the near-perfect overlap between the main and evaluation curves confirms that our cost-driven decision logic replicates well on held-out data.

Figure 2D–E shows confusion matrices at representative ζ values. At negative ζ, more than 90% of true IMC and ICU cases are assigned to lower-acuity settings (under-triage), illustrating the clinical risk when the objective function heavily penalizes resource utilization. As ζ increases toward 0.0, correct regular-ward classifications increase, and IMC classification performance begins to recover. At ζ =  + 1.0, class-wise correct allocation is ≥ /≈ 50% in the development cohort (with comparable behavior in the evaluation cohort; Fig. 2D–E). Between these extremes, performance changes only modestly across a plateau (approximately ζ = 0.5–1.5). We therefore performed a fine-grained sweep in this interval (Δζ = 0.025) to characterize the under- versus over-triage trade-off (Fig. 2C).

Across bootstrapped resamples, the median inflection-point estimate was ζ = 0.975 (Fig. 2B), which was used as the operating point for subsequent analyses. The relevant performance metrics are listed in Table 4, and their dynamics are depicted in supplementary Fig. S3. Because the evaluation cohort contained few ICU-level events (6/81), class-specific ICU metrics are sensitive to small absolute-count changes. We therefore summarize external-cohort performance across 25 independently trained models (mean ± SD and 95% CI of the mean; Supplementary Table S1).

**Table 4:** Main performance characteristics for ζ = 0.975 (mean ± SD).

	Main dataset	Evaluation dataset
AUCµ	0.67 ± 0.03	0.60 ± 0.04
F1_w_	0.49 ± 0.03	0.44 ± 0.06
F1 _regular ward_	0.58 ± 0.04	0.53 ± 0.08
F1 _IMC_	0.32 ± 0.05	0.26 ± 0.12
F1 _ICU_	0.36 ± 0.05	0.22 ± 0.06

### Relative importance of prognostic features

Understanding which features most influence triage decisions is crucial for model transparency and clinical trust. To this end, we employed two complementary metrics—GAIN and SHAP—to identify the variables driving acuity assignments. To this end, we evaluated how the decision is determined at our optimal ζ = 0.975 and how these importances evolve as ζ diverges from its optimal value.

**Fig. 3:**
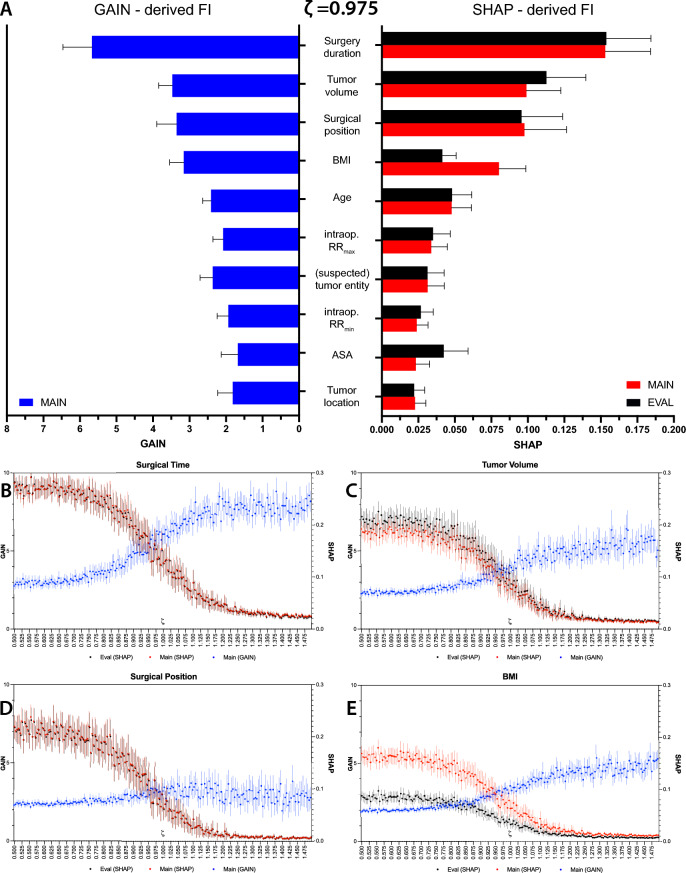
Feature importance of Neuro-TACTIC and its dependence on the cost-sensitivity parameter ζ. (**A**): Global importance of the ten most influential features in the development and validation cohorts (n = 1072, n = 81) at the optimal ζ = 0.9750. GAIN-based importance values (blue bars, plotted to the left) are computed on each training split during cross-validation and are therefore only available for the main dataset; features are sorted in descending order of mean GAIN. Mean absolute SHAP values are shown for both the development cohort (red bars) and the independent evaluation cohort (black bars), capturing each feature’s average contribution to individual predictions across all samples. Surgery duration ranks highest by both metrics, followed by tumor volume, surgical position, body mass index (BMI), and patient age. Error bars denote mean ± s.d. across 25 cross-validation folds. (**B–E**) Evolution of feature importance across ζ values between 0.5 and 1.5 (the “tuning” region) for the four top-ranked variables: surgical duration (**B**), tumor volume (**C**), Surgical position (**D**), and BMI (**E**). In each panel, red circles and solid lines indicate GAIN-based importance, while blue squares and dashed lines represent mean absolute SHAP values, both averaged over 25 cross-validation runs (error bars represent mean ± 1 s.d.). As ζ increases (greater emphasis on avoiding under-triage), gain importance for each feature rises—indicating that higher ζ settings drive the model to rely more heavily on these predictors to escalate care—whereas SHAP importance peaks at lower ζ, reflecting that when resource cost is prioritized, small changes in these features more strongly influence individual acuity assignments.

The GAIN‐derived importance scores (Fig. 3A, left side, blue bars) quantify the total information gain each feature contributes to the model’s splits. Operative duration was the predominant predictor by both GAIN and SHAP, followed by tumor volume and surgical position. As operative duration is definitively known only after completion of surgery, the present model is primarily applicable to immediate postoperative disposition rather than preoperative scheduling; future iterations will therefore evaluate reduced feature-set (preoperative-only) variants. Surgical position, BMI, and patient age comprise the top five factors, followed by intraoperative ranges of relative risk, suspected tumor entity, and ASA status. Importantly, rankings are highly stable across the repeated 50 bootstrap samples.

SHAP‐derived importance scores (Fig. 3A, right side, red for the main dataset, black for the evaluation set) confirm these findings. SHAP-features are a measure of the average absolute change in the model’s output attributable to each feature. Again, surgery duration leads, driving roughly 0.15 units of predicted acuity shift on average, with tumor volume and surgical position close behind. BMI and age occupy an intermediate tier, while tumor location and ASA status have comparatively marginal effects. Crucially, the similarity between the main and evaluation datasets confirms that the model generalizes seamlessly to the held‐out data.

To assess the changes in feature importance over the ζ continuum, we derived the respective GAIN and SHAP values from 0.5 to 1.5 in increments of 0.025 for the four features most important at ζ = 0.975 (Fig. 3, bottom panels). All four features exhibit a characteristic “crossover” behavior. For example, the importance of surgery duration in GAIN terms steadily increases with ζ: when harm-costs dominate, the model places more emphasis on procedural time to decide whether to escalate care. Conversely, its SHAP importance peaks in the negative‐ζ regime, where small changes in duration can tip the scale from ward to IMC or ICU under strict resource constraints. Tumor volume and surgical position display the same inverse relationship between GAIN and SHAP curves, while BMI’s influence remains relatively muted overall but still shifts subtly with ζ.

## Discussion

Neuro-TACTIC introduces a cost-sensitive, three-tiered approach to postoperative neurosurgical triage. By defining a relative cost matrix that encodes personnel expenditure for regular wards (10:1 nurse: patient), IMC (4:1), and ICU (2:1) settings, and combining it with a harm-cost matrix reflecting event rarity. The model’s ζ parameter smoothly navigates between under- and over-triage priorities. The ζ parameter yields a plateau (≈0.5–1.5) where IMC assignments peak. In our current dataset, we consistently identified ζ = 0.975 as a good trade-off between resource conservation and under-triage risk, and fixed this value for all subsequent analyses.

All previous models were based on a binary ICU-versus-non-ICU framework and therefore overlook the growing middle tier of intermediate care^1,2,10,22^. For example, Hanak and colleagues applied a logistic regression model to 400 elective craniotomy cases and found that only age and diabetes predicted postoperative ICU requirements, without providing any gradation in intermediate monitoring need^22^. Similarly, Munari et al. developed a simple point score combining ASA-PS, tumor volume, and surgery duration and reported an ROC-AUC of 0.774 for ICU admission, whereas CranioScore yielded an ROC-AUC of 0.70. Follow-up validations on novel cohorts reproduced discriminations in the 0.65–0.75 range, yet both tools collapsed regular-ward and IMC candidates into a single low-acuity category.

Even ML efforts that moved beyond linear models remained binary, consistently outperforming regression models^10,23^, but they too produced only ICU versus non-ICU recommendations^10,23^. Additionally, none of these prior approaches included a transparent lever to adjust sensitivity versus specificity in accordance with local staffing ratios or definitions of which events truly mandate ICU care. By contrast, Neuro-TACTIC’s three-level output explicitly accommodates intermediate care and introduces ζ as a cost-sensitive tuning parameter.

Essentially, our approach formalizes what expert clinicians implicitly do: consider placing every patient in the ICU as the upper bound of safety (ζ <  < 1), and then use a cost-sensitive framework to reduce ICU admissions to the minimum necessary. Notably, because our clinical setting does not routinely require ICU admission, the patients included in this study were classified as “*high-risk*” by experienced clinicians.

In the retrospective cohorts analyzed, the model produced assignment distributions consistent with reduced ICU allocation at selected cost settings. These findings demonstrate the feasibility of cost-sensitive triage modeling. Routine ICU care for elective craniotomy has been shown to consume multiple times the staff and cost of ward or IMC care, yet benefits only a small minority of patients ^3,4,24,25^. A recent study examined 1,070 consecutive craniotomy patients; 674 were monitored overnight in an ICU, and 396 were observed postoperatively in a PACU before transfer to the regular ward, reporting virtually identical incidence rates of any relevant event within 24 h postoperatively (4% in ICU, 2.8% in PACU). In our cohorts, the overall rate of ICU-level events was substantially higher (12.4% in the main dataset and 7.4% in the evaluation set), reflecting either a different case mix (e.g., markedly more frequent infratentorial surgeries in our collective) or more inclusive event monitoring, mirroring similar rates of postoperative ICU-level events reported by others^24^.

A hospital that classifies minor neurological deficits as ICU-level events can increase the corresponding under-triage penalty, whereas a facility with constrained ICU capacity can increase the resource-cost weight associated with ICU allocation. Importantly, despite measurable case-mix differences between the development and evaluation cohorts, the model exhibited similar ζ-dependent allocation trajectories and operating-point behavior across datasets. At ζ = 0.975, assignment distributions were comparable between cohorts and reflected lower ICU allocation at this operating point. These findings suggest stable model behavior under the observed cohort shift, while acknowledging that ICU-class performance estimates in the evaluation cohort remain imprecise due to the low number of ICU events. Performance in the independent evaluation cohort was modest for the IMC and ICU classes, which is expected given marked class imbalance and only six ICU-level events. Nonetheless our findings support the feasibility and stability of the cost-sensitive multiclass framework, while they do not justify autonomous clinical deployment at this stage. Because ζ explicitly encodes institution-specific trade-offs, there is no single universal AUC/F1 threshold for implementation; rather, prospective translation would require pre-specified safety/utility targets agreed with stakeholders (e.g., constraints on ICU under-triage) and evaluation against current standard practice.

To understand which factors drive Neuro-TACTIC’s triage recommendations, we assessed two complementary measures of feature importance across our XGBoost models (GAIN and SHAP). Gain- and SHAP-based analyses both ranked operative duration, tumor volume, and surgical position as top predictors (Fig. 3A). Emphasizing these features is consistent with numerous studies assessing risk factors for postoperative ICU events (for a summary, see Table 5)^1,2,10,22–24,26^.

**Table 5:** Qualitative comparison of whether key predictors identified by Neuro-TACTIC were evaluated or reported in prior studies of postoperative monitoring/ICU disposition after elective craniotomy. “X” indicates the variable was explicitly included or reported; blank indicates not reported.

References	Methodology	n	Surgical duration	Tumor volume	Surgical position	BMI	AGE	Suspected entity	ASA	Tumor location
Van Nftrik et al.^23^	Gradient boosted trees, not further specified	668		X	X	X	X	X		X
Hanak et al.^22^	Multivariable logistic regression	400					X			
Neumann et al.^10^	XGBoost derived SHAP values	1000	X	X	X	X	X	X	X	
Ehlers et al.^26^	Logistic regression	282	X							
Cinotti et al.^2^	Multivariable logistic regression	1094	X							
Munari et al.^1^	Multivariable logistic regression	420	X	X					X	
Kurz et al.^24^	Multivariate logistic regression	860	X				X	X	X	

## Limitations

Several limitations warrant careful consideration. First, the dataset underlying the computational framework was derived from patients who had already been pre-allocated to the ICU by the treating surgeon, representing a clinically enriched population rather than an unselected elective craniotomy cohort. Such enrichment may influence baseline ICU/IMC event frequencies and potentially affect discrimination characteristics. However, published series of elective craniotomy report substantial variability in postoperative ICU-level event rates, ranging from low single-digit unplanned ICU admissions in selectively monitored PACU cohorts to rates in the low teens for adverse events requiring intensive care intervention^3,27,28^. Accordingly, while our cohort reflects a higher-risk population, the observed ICU-level event frequencies remain within ranges described in the literature. Nonetheless, extrapolation to broader, lower-risk elective populations should be undertaken cautiously.

In addition, the single-center, retrospective design may limit generalizability, as local practice patterns (such as criteria for IMC admission versus ward observation), perioperative care protocols, and definitions of “ICU-level” events vary across institutions and healthcare systems. The model’s decision boundaries and ζ-derived trade-offs may therefore require recalibration before application elsewhere; however, such recalibration is facilitated by the inclusion of ζ. Neuro-TACTIC should therefore be interpreted as a methodological proof-of-concept rather than a clinical decision rule.

Second, the relative rarity of true ICU-level complications (class imbalance) poses challenges to robustly estimating sensitivity. Although cross-validation and our held-out evaluation cohort (n = 81) yielded similar assignment rates, the small absolute number of positive events means that confidence intervals for each individual adverse outcome remain wide. This limits our ability to explore more granular event subtypes (e.g., reintubation vs. catecholamine use), each of which may warrant its own risk profile and cost parameter. For meaningful external validation of ICU-class performance, future cohorts should include substantially larger numbers of ICU-level events (ideally ≥ 50), to allow reasonably precise estimation of ICU sensitivity/F1; this will likely require prospective multicenter data collection.

Third, while SHAP and gain-based analyses reliably identify the most influential features, the complex interactions are not immediately transparent to end users. Further work is therefore needed to translate these insights into intuitive decision aids.

Finally, our current framework focuses exclusively on preoperative and intraoperative variables to predict immediate postoperative care needs. It does not account for dynamic changes during the recovery period that might further refine acuity assignments. Because operative duration is only fully known at the end of surgery, the current Neuro-TACTIC instantiation is primarily applicable to immediate postoperative disposition decisions (ward vs IMC vs ICU) rather than preoperative scheduling. This is a limitation, as earlier risk stratification would be desirable for operational planning and capacity management. Future iterations will therefore evaluate reduced feature-set variants (e.g., preoperative-only models) to enable preoperative scheduling decisions, consistent with our proof-of-concept positioning.

## Conclusions

Neuro-TACTIC introduces a three-tier, cost-sensitive modeling framework for postoperative neurosurgical triage that explicitly incorporates intermediate care alongside regular ward and intensive care unit assignment. By encoding relative resource-use and harm considerations into the tunable parameter ζ, the framework enables systematic exploration of trade-offs between under- and over-triage in retrospective data. In the cohorts analyzed, a ζ value of 0.975 was selected as the operating point, yielding stable assignment behavior in both the development (n = 1,072) and independent evaluation (n = 81) datasets.

Across models, operative duration, tumor volume, surgical position, body mass index, and patient age emerged as the most influential predictors, consistent with established risk factors reported in prior studies. This concordance supports the framework’s interpretability and its alignment with known clinical determinants of postoperative acuity.

The present study is intended as a methodological proof of concept. Prospective, multicenter validation, incorporation of dynamic perioperative data, and evaluation of clinical and economic outcomes will be necessary to determine the utility of Neuro-TACTIC before any clinical application.

## Supplementary Information

Supplementary Information 1.

Supplementary Information 2.

## Data Availability

Due to institutional and privacy regulations, the clinical dataset cannot be made publicly available. De-identified data may be available upon reasonable request and pending institutional approval.

## Code availability

The code used to develop and evaluate the Neuro-TACTIC framework (including the cost-sensitive XGBoost objective, ζ-sweep routines, model training/evaluation scripts, and figure generation) will be made publicly available via the University of Heidelberg’s institutional research data repository (HeiData) in a versioned release upon publication and can be accessed at http://neuro-tactic.predict-icu.de. Where applicable, the release will include a minimal example pipeline and documentation to facilitate reuse by other centers.
