# Supplementary material for: A cost-sensitive multiclass machine learning framework for postoperative neurosurgical triage (Neuro-TACTIC)
Source: Sci Rep. 2026 Mar 24;16:9847. doi: 10.1038/s41598-026-45092-1 (PMC13018626; doi:10.1038/s41598-026-45092-1)
Supplement: Supplementary file 2 — Supplementary Information 2. [file 41598_2026_45092_MOESM2_ESM.docx]

**Supplementary:**

**Supplementary Methods:**

## Cost-Matrix and weighted loss function

Taking the relative staffing needs as a proxy for clinical cost, we defined a relative cost matrix RC. For a typical ICU setting, we assumed a 2:1 ratio, a typical IMC ratio of 4:1, and on a regular ward, a 10:1 patient-to-staff ratio in a regular ward. RC is therefore defined as a row-normalized matrix:

$$RC=\begin{matrix} 0 & \frac{r_{1}}{r_{0}} & \frac{r_{2}}{r_{0}} \\ 0 & 0 & \frac{r_{2}}{r_{2}} \\ 0 & 0 & 0 \end{matrix} , normalized as RC\leftarrow\frac{\mathrm{RC}}{\sum_{i,j} \mathrm{RC}_{i,j}}$$

r_0_, r_1_, r_2_ are the relative cost weights for each class, chosen based on the personnel expenditure for the regular ward, IMC, and ICU, respectively

$$r= \left[ \frac{1}{10},\frac{1}{4},\frac{1}{2} \right]$$

The Harm Cost (HC) matrix is computed as the inverse frequency of the class distribution in the training dataset. n0, n1, n2 represent the respective numbers of regular ward, IMC, and ICU patients, respectively, within the entire dataset N.

$$HC=\begin{matrix} 0 & 0 & 0 \\ \frac{n_{1}}{N} & 0 & 0 \\ \frac{n_{0}+n_{1}}{N} & \frac{n_{0}}{N} & 0 \end{matrix} , normalized as RC\leftarrow\frac{\mathrm{RC}}{\sum_{i,j} \mathrm{RC}_{i,j}}$$

To find the optimal relation between relative cost and potential harm, we introduce a factor $\zeta$ to define the final cost matrix CM as

$${CM}_{\zeta}=RC\cdot e^{-\zeta}+HC\cdot e^{\zeta}$$

To find an optimal $\zeta$value, we incrementally modified $\zeta$from -5 to 5 in steps of 0.01 and repeated the cross-validation $\zeta$ 50 times.

**Practical calibration of ζ**

The tunable parameter ζ is intended to be calibrated using institution-specific retrospective data prior to prospective evaluation. A practical calibration workflow comprises: (i) defining local event labels (ward/IMC/ICU) consistent with institutional practice and documentation; (ii) estimating class frequencies and ensuring sufficient event counts to support stable performance estimation; (iii) performing an initial coarse ζ sweep (e.g., −5 to +5) to identify the plateau region in which allocations transition smoothly; (iv) refining within the plateau using smaller ζ increments (e.g., 0.025) while monitoring under-triage and over-triage components; and (v) selecting an operating point under pre-specified constraints agreed with local stakeholders (e.g., maximum acceptable ICU under-triage) and resource priorities.

Internal validation should be performed using repeated cross-validation and/or bootstrap resampling to assess stability of allocation trajectories and error decomposition across ζ. Sensitivity of model behavior to ζ can be quantified by reproducing the allocation curves and under-/over-triage decomposition as functions of ζ, analogous to Figure 2A–C and Supplementary Fig. S3, and by confirming that candidate operating points yield consistent behavior across resamples.

Data volume requirements depend on outcome rarity. Because ICU-level events are often infrequent, meaningful calibration and external validation of ICU-class performance requires adequate ICU-event counts; as a pragmatic benchmark, cohorts with on the order of **≥50 ICU-level events** are desirable to enable reasonably precise estimation of ICU sensitivity/F1. If event counts are lower, ζ selection should be interpreted as exploratory and results should be reported with appropriate uncertainty and framed as hypothesis-generating, motivating multicenter aggregation or prospective data collection.

**Supplementary Figure S1: Confusion matrices for the main cohort across representative ζ settings.**
Each panel shows a 3×3 confusion matrix of actual (rows) versus predicted (columns) postoperative acuity assignments—regular ward (RR), intermediate-care unit (IMC), and intensive care unit (ICU)—for the main dataset (n = 1,072). Within each cell, the top number is the percentage of patients, and the number in parentheses is the count. Panels are arranged in ascending order of the cost–harm weighting parameter ζ (–1.95, –1.45, –0.95, –0.45, +0.05, +0.55, +1.05, +1.55, +2.05, +2.55), illustrating how increasing ζ shifts the model’s tendency from under-triage (favoring RR) toward over-triage (favoring ICU) with an intermediate peak in correct IMC classifications around ζ≈+0.5. Confusion matrices show mean cell counts across repeated cross-validation folds; counts, therefore, may not equal the raw cohort event counts.

**Supplementary Figure S2:** **Confusion matrices for the evaluation cohort across representative ζ settings.**
Each panel displays a 3×3 confusion matrix of actual (rows) versus predicted (columns) postoperative acuity assignments—regular ward (RR), intermediate-care unit (IMC), and intensive care unit (ICU)—for the independent evaluation dataset (n = 81). Within each cell, the top number indicates the percentage of patients, and the number in parentheses shows the count. Panels are ordered by increasing cost–harm weighting parameter ζ (–1.95, –1.45, –0.95, –0.45, +0.05, +0.55, +1.05, +1.55, +2.05, +2.55), illustrating how rising ζ shifts predictions from predominant under-triage (favoring RR) through an intermediate peak in correct IMC assignments to eventual over-triage (favoring ICU). Confusion matrices show mean cell counts across repeated cross-validation folds; counts, therefore, may not equal the raw cohort event counts.

 **Supplementary Figure S3:**

**(A)** Cost-weighted multiclass ROC surface area (AUCµ) plotted against ζ (0.5–1.5) for both the main (n = 1,072; blue) and held-out evaluation (n = 81; orange) datasets, showing nearly overlapping curves and a rising optimum near ζ≈0.975. **(B)** Class-specific F₁ scores in the main dataset as functions of ζ: regular-ward (solid line), IMC (dashed), and ICU (dotted), illustrating how each acuity class’s precision–recall balance shifts with varying cost sensitivity. **(C)** Corresponding class-specific F₁ scores in the evaluation cohort—ICU (dotted), regular-ward (solid), and IMC (dashed)—demonstrating generalization of the main-cohort performance trends. **Abbreviations:** ζ, relative cost–harm weighting parameter; IMC, intermediate-care unit.

| **Class** | **Metric** | **Mean** | **Std. Dev.** | **95% CI of the mean** |
| --- | --- | --- | --- | --- |
| **ICU** | sensitivity (recall) | 0.4467 | 0.1908 | 0.3679 – 0.5254 |
|  | precision (PPV) | 0.1559 | 0.0395 | 0.1396 – 0.1723 |
|  | F1 score | 0.2250 | 0.0607 | 0.1999 – 0.2500 |
| **IMC** | sensitivity (recall) | 0.3410 | 0.1933 | 0.2612 – 0.4207 |
|  | precision (PPV) | 0.2434 | 0.1279 | 0.1906 - 0.2962 |
|  | F1 score | 0.2779 | 0.1477 | 0.2169 – 0.3388 |
| **Regular Ward** | sensitivity (recall) | 0.4448 | 0.1088 | 0.3999 – 0.4898 |
|  | precision (PPV) | 0.7114 | 0.06182 | 0.6859 – 0.7370 |
|  | F1 score | 0.5390 | 0.08377 | 0.5044 – 0.5736 |
|  |  |  |  |  |

**Supplementary Table S1. External evaluation cohort class-wise performance across 25 independently trained models (ζ = 0.975).**
Performance metrics were computed on the independent evaluation cohort (n = 81) by applying each of the 25 models obtained from the repeated cross-validation procedure on the development cohort to the same evaluation cohort. Values are reported as mean, standard deviation (SD), and 95% confidence intervals (CI) of the mean across these 25 model evaluations.
